# Supporting the education evidence portal via text mining

**DOI:** 10.1098/rsta.2010.0152

**Published:** 2010-08-28

**Authors:** Sophia Ananiadou, Paul Thompson, James Thomas, Tingting Mu, Sandy Oliver, Mark Rickinson, Yutaka Sasaki, Davy Weissenbacher, John McNaught

**Affiliations:** 1 School of Computer Science and National Centre for Text Mining, University of Manchester, 131 Princess Street, Manchester M1 7DN, UK; 2 EPPI-Centre, Social Science Research Unit, Institute of Education, University of London, 20 Bedford Way, London WC1H 0AL, UK

**Keywords:** text mining, term extraction, document classification, document clustering

## Abstract

The UK Education Evidence Portal (eep) provides a single, searchable, point of access to the contents of the websites of 33 organizations relating to education, with the aim of revolutionizing work practices for the education community. Use of the portal alleviates the need to spend time searching multiple resources to find relevant information. However, the combined content of the websites of interest is still very large (over 500 000 documents and growing). This means that searches using the portal can produce very large numbers of hits. As users often have limited time, they would benefit from enhanced methods of performing searches and viewing results, allowing them to drill down to information of interest more efficiently, without having to sift through potentially long lists of irrelevant documents. The Joint Information Systems Committee (JISC)-funded ASSIST project has produced a prototype web interface to demonstrate the applicability of integrating a number of text-mining tools and methods into the eep, to facilitate an enhanced searching, browsing and document-viewing experience. New features include automatic classification of documents according to a taxonomy, automatic clustering of search results according to similar document content, and automatic identification and highlighting of key terms within documents.

## 1. Introduction

In education, as in many other professions, the Internet is becoming an increasingly important tool to provide the evidence required for practice and policy making. One of the problems faced by both professionals and lay people is that research findings can be dispersed among multiple sources, meaning that considerable time often has to be spent locating relevant resources. In response to this, the UK Education Evidence Portal (eep) has been created, which draws on documents from a range of reputable UK sources. By making these collections searchable from a central point of access, the portal aims to revolutionize work practices for the education community. It has its roots in a growing concern that interventions in people’s lives should be informed by the best available evidence. Currently, education policy makers and practitioners do not make as much use of research evidence as they might; this is partly because it is fragmented, difficult to find and is sometimes written in inaccessible language. The eep therefore provides a search engine that enables users to search the contents of the websites of 33 organizations that publish freely accessible educational evidence, prioritizing documents and summaries that are suitable for the busy professional. It includes resources from organizations such as the Department for Children, Schools and Families, the National Foundation for Education Research, the Institute of Education, London, the General Teaching Council for England, the Higher Education Academy, Her Majesty’s Inspectorate of Education, Ofsted and the Scottish Executive.

While the eep provides a convenient point of access to relevant documents, the amount of data contained within its database is still very large—based on web-crawl results of the website content of the 33 organizations, the eep dataset contains over 500 000 documents and, owing to the vast amounts of new scientific data that are being produced in the context of e-Science (Hey & Hey 2006), this number is likely to continue to grow significantly. As the number of documents increases, traditional search-engine techniques, in which a user’s keyword search simply returns a potentially long list of documents containing the keywords, are becoming increasingly inefficient because the user has to spend valuable time determining which documents contain information that is relevant to their needs.

Developments in social science research, such as systematic reviewing, make the need for more efficient searching even more critical. Systematic reviews can take more than a year to complete, with up to half of that time being spent searching and screening hits. This is problematic because policy makers and practitioners often need to know the state of research evidence over a much shorter time scale than current methods allow. This may result in situations where research evidence is not used at all, with consequential dangers for people affected by policies or practices developed in the absence of a firm evidence base (Chalmers 2003). As a result of such difficulties, text-mining techniques are receiving increasingly more attention (Ananiadou *et al.* 2009).

By automatically retrieving knowledge from unstructured text, text-mining techniques can provide enhanced views of search results, which permit users to perform more focussed searches than previously possible, and allow them to locate relevant information within the retrieved documents in a more timely and efficient manner. These additional views include the automatic classification of documents according to pre-defined subjects in a custom-built, domain-specific taxonomy, automatic clustering of search results according to the most prevalent phrases contained within them and highlighting of key terms within documents, allowing their content to be skimmed more easily.

The aim of the Joint Information Systems Committee (JISC)-funded ASSIST project has been to investigate the benefits of text mining in the social science disciplines, in which textual information constitutes an important source of knowledge. Importantly, the project has adopted a user-centred design–build–evaluate approach, ensuring close interaction between developers and users from the earliest stages of the design, through to implementation and evaluation. This article describes the prototype web interface (http://nactem.mc.man.ac.uk/ASSIST-EPPI/) that has been developed as part of the ASSIST project, in order to demonstrate how the search facilities of the eep portal could be expanded and enhanced through the inclusion of a number of text-mining methods, such as those introduced above.

The remainder of the paper is organized as follows. In §2, an overview of text mining is provided, together with a brief description of the particular text-mining technologies that have been integrated into the ASSIST prototype interface. Section 3 explains, in more detail, the overall searching and browsing functionalities of the interface. Section 4 covers the enhanced indexing process employed by the interface and the benefits that this brings. In §5, the automatic classification of documents according to a domain-specific taxonomy is described, while §6 explains the automatic document-clustering strategy. In §7, we detail our user-centred evaluation framework and provide some emerging results from the evaluation of the interface. Finally, in §8, we provide concluding remarks, together with directions for future work.

## 2. Text mining

The primary goal of text mining is to discover knowledge that is hidden in text, and to present this distilled knowledge to users in a concise form. Text mining is a variation of data mining in that, while data mining discovers knowledge from *structured* data (Hearst 1999), text mining has the aim of discovering and extracting knowledge from *unstructured* data, i.e. free text. Text mining thus involves extra processing steps in order to locate, extract and structure relevant information from texts prior to the knowledge discovery (i.e. data-mining) step. The advantage of text mining is that it enables researchers to collect, maintain, interpret, curate and discover knowledge needed for research or education, in an efficient and systematic way (Ananiadou & McNaught 2006).

Text mining comprises three major activities,

1. *Information retrieval*. Gathering of relevant texts.
2. *Information extraction*. Looking within the retrieved texts to identify, extract and structure a range of specific types of information or facts.
3. *Data mining*. Finding associations among the pieces of information extracted from many different texts.

Thus, text mining can dramatically reduce the amount of work required by the user—instead of being presented with potentially tens of thousands of documents to sift through and comb for relevant knowledge, text mining offers the possibility of automatically extracting and presenting to the user precise facts retrieved from relevant documents. Furthermore, interesting associations may be found among disparate extracted facts, leading to the discovery of new or unsuspected knowledge.

Within the ASSIST project, several text-mining technologies have been used to support the tasks expected by users of the eep. Each technology either provides new features, or enhances the existing components of the education portal. The main technologies employed are as follows:

1. *Automatic classification of documents*. Documents are indexed according to a domain-specific taxonomy (see §5), allowing users to browse documents related to particular taxonomy terms, as an alternative to the more traditional free-text search. The hierarchical structure of the taxonomy allows searches to be more or less specific, according to users’ individual requirements.
2. *Automatic clustering of search results*. Rather than relying on standard ranking of documents using relative term frequency, results of queries are made more manageable for the user by automatic clustering of related documents.
3. *Identification of key terms*. Documents are automatically analysed with the TerMine tool (http://www.nactem.ac.uk/software/termine/), developed at the National Centre for Text Mining (NaCTeM). This facilitates highlighting of key terms within documents that can help to characterize their content, as well facilitating enhanced search functionality based on the terms.
4. *Advanced search capabilities*. Additional operators may be specified within free-text searches to allow searching on document metadata, such as titles, author names and keywords. This allows, for example, only those documents with a particular author to be retrieved by a search.

## 3. Document searching and browsing

The enhanced eep prototype makes use of document indexing provided by the ASSIST framework. This framework builds upon an existing modular search platform developed during the ASSERT project (Ananiadou *et al*. 2009), into which different text-mining tools can plugged. The searching and indexing technology is based on Apache Lucene (Hatcher & Gospodnetic 2004; http://lucene.apache.org/).

As with all search engines, documents must be indexed prior to searching by end users. In addition to standard indexing, the ASSIST platform includes enhanced indexing techniques, which are described in more detail in §4.

Once indexed, documents may be searched using two main methods,

1. Google-style free-text queries that may include the logical operators AND and OR, as well as the wildcard character *. Custom operators (such as AUTHOR or TITLE) also allow searches to be performed on either metadata extracted from documents (see §4) or terms identified by TerMine.
2. Browsing the custom-built, domain-specific taxonomy of relevant subjects (see §5). Selecting a term from taxonomy causes all documents relevant to that subject to be displayed. Once a taxonomy term has been selected, free-text searching can be carried out within the retrieved set of documents, allowing the search to be refined further.

Regardless of the search method used, the returned results are automatically assigned to clusters that are generated on demand according to the most prevalent phases in the documents (see §6). The provision of clusters means that users are more easily able to ‘drill down’ to the documents of most interest to them, without having to sift through a single long list of documents.

**Figure 1.**
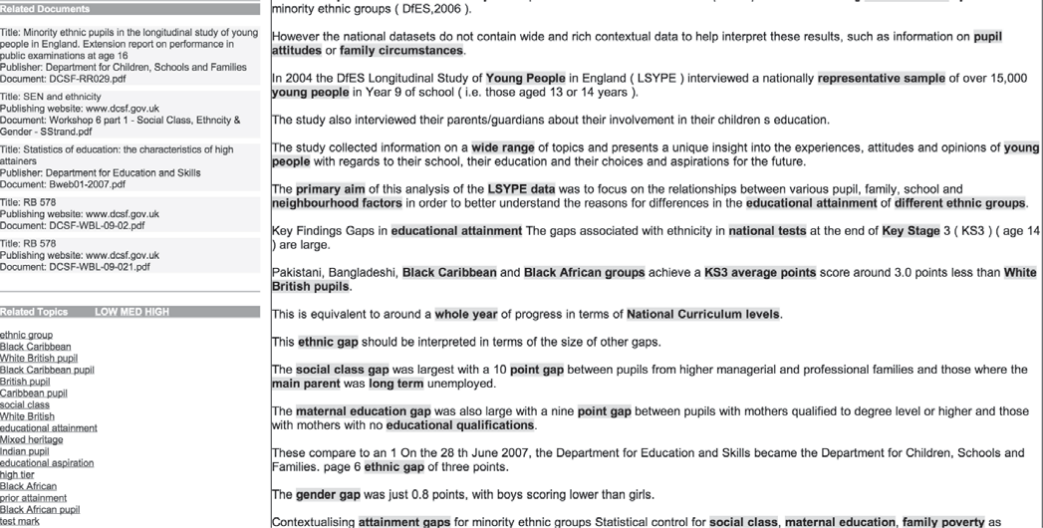
Document analysed with TerMine.

Finally, the user has two ways of finding out more about the document. Firstly, it is possible to view the document in its original format (e.g. Portable Document Framework; PDF). Secondly, there is the option to view metadata regarding the document, including author and date of publication, together with a list of all terms assigned from the taxonomy, which provide a quick overview of the topics covered by the document. This second view of the document also allows the *Text Mining Analysis* of the document to be displayed, consisting of a plain text version of the document, with all TerMine-identified terms highlighted within the text (see figure 1).

Users of the interface have noted that the highlighting of terms in this way supports the ability to quickly skim through documents for relevant sections. *The* *Text Mining Analysis* view includes two additional features that are designed to help users to browse related documents. Firstly, *Related Documents* displays the titles of a set of documents most closely related to the current one, based on the cooccurrence of terms. Secondly, *Related Topics* displays a hyperlinked list of all TerMine-identified terms found in the document. Terms are ranked according to their *termhood*, i.e. their importance in characterizing the document (see §3*a*). Clicking on a term will cause a new search to be carried out, based on that term.

### (a) TerMine

The automatic extraction of the multi-word terms that characterize a document’s content is carried out using NaCTeM’s TerMine tool. The advantages of this tool are that it is domain independent and does not rely on dictionaries. It exploits both linguistic and statistical information in order to identify candidate terms and rank them according to their *termhood* or C-Value score (Frantzi *et al*. 2000). Firstly, linguistic information (i.e. parts of speech) is used to determine which types of word sequences (e.g. combinations of adjectives and nouns) could potentially be terms. An optional stopword dictionary can be applied. Secondly, candidate terms are extracted based on the following statistical information:

— the total frequency of occurrence of the candidate string in the corpus,
— the frequency of the candidate string as part of other longer candidate terms,
— the number of these longer candidate terms and
— the length of the candidate string (number of words).

These pieces of information are involved in the calculation of an individual score (the C-Value) for each candidate term. The list of candidate terms is ordered according to their C-Value scores, and all the candidates above a certain threshold are designated as terms for the document. Within the eep interface, is possible to set the threshold at three different levels (i.e. *LOW*, *MED* and *HIGH*) when viewing particular documents.

## 4. Enhanced indexing

In addition to standard indexing, ASSIST adds two extra types of indices to Lucene to provide enhanced searching capabilities.

1. *Document metadata*. These correspond to existing annotations that are encoded within the structure of the document. i.e. author, title, subject (manual summary of the document content) and keywords (manually selected keywords describing the document content). Extracting this information allows advanced searches to be carried out using the values of these fields, e.g. searching for all documents with particular words in their titles.
2. *Automatic term extraction using TerMine*. Users can restrict their search to find only those documents in which their search terms have been identified as key terms by TerMine. This extraction mechanism also allows the identification of ‘Related Documents’ in the *Text Mining Analysis* view, as described in the previous section.

The main stages of the indexing process are as follows:

— *Document conversion.* Lucene requires documents to be in plain-text format. Third-party components have been adopted for the conversion of each document type (i.e. PDF, Hyper Text Markup Language (HTML), Extensible Markup Language (XML), Microsoft Word) to plain text, with some modifications to allow metadata to be recovered from the original document structure.
— *Document annotation*. The annotations of interest to end users of the interface are those produced by TerMine. However, as a prerequisite to running TerMine over a document, linguistic pre-processing firstly has to be carried out. This consists of the following steps:
  - *Tokenization.* Breaking the text into individual words and other basic units such as punctuation, numbers, dates, formulae, Uniform Resource Locators (URLs)
  - *Part of speech* (*POS*) *tagging.* Carried out by the Genia tagger (Tsuruoka *et al.* 2005; http://www-tsujii.is.s.u-tokyo.ac.jp/GENIA/tagger/).
  - *Sentence splitting.* Carried out on the basis of punctuation patterns and other lexicographic evidence (http://text0.mib.man.ac.uk:8080/scottpiao/sent_detector).

## 5. Automatic classification of documents

### (a) The Education Evidence Portal taxonomy

In order to create a taxonomy suitable for browsing documents in the eep database, existing educational taxonomies were first evaluated, but determined to be too narrow in scope. A new taxonomy was therefore developed during 2008/2009 by the eep consortium with the intention of

— enabling users to browse through content by a hierarchical directory of broad topics,
— giving users an overview of all the topics covered and the volume of resources,
— being a framework for introducing new topics and
— using the topics as the basis of an alerting service in the future.

The work was led by the Department for Children, Schools and Families, and included multiple consultations with eep partners and the wider education community. Two conceptual structures were drafted and evaluated by the group: one organized by educational level and the other by topic. The topic-based structure was eventually chosen, as it was felt that this organization encourages users to retrieve information outside their immediate level, which fosters learning outside traditional sources. In order to support the retrieval of information according to educational level when required, an additional filter for this was added.

The development work then turned to a wider consultation via a web-based simulation hosted on the portal, resulting in more than 180 responses. The wider evaluation brought useful suggestions for modifications and also identified existing sets of terms to be used within the taxonomy (e.g. the Joint Academic Coding system (JACS) for higher education). At the same time, a detailed review of eep content on the British Education Index (BEI) was carried out in order to evaluate the extent to which the taxonomy described existing eep documents.

In light of the above, the taxonomy was revised and then deployed to the BEI in Leeds. The BEI modified its systems in order to support the new taxonomy, and the team then indexed the 3000+ eep documents using the taxonomy.

The final taxonomy consists of 108 concept categories. The taxonomy is organized into broad topics with a shallow hierarchy, owing to the specific purpose for which it has been designed. Rather than being highly detailed and exhaustive, containing thousands of terms (like, for example, the British Education Thesaurus), the intention is that the taxonomy complements the other tools within the eep.

The taxonomy has a fairly flat structure, with usually only one level beneath the top-level terms. At the top level, the taxonomy consists of

— curriculum, subjects and skills,
— teaching and learning,
— performance, assessment and quality improvement,
— careers, work experience and employment,
— management, governance and finance,
— teachers and staff,
— families, community and society,
— care welfare and behaviour and
— research methods and use of evidence.

**Figure 2.**
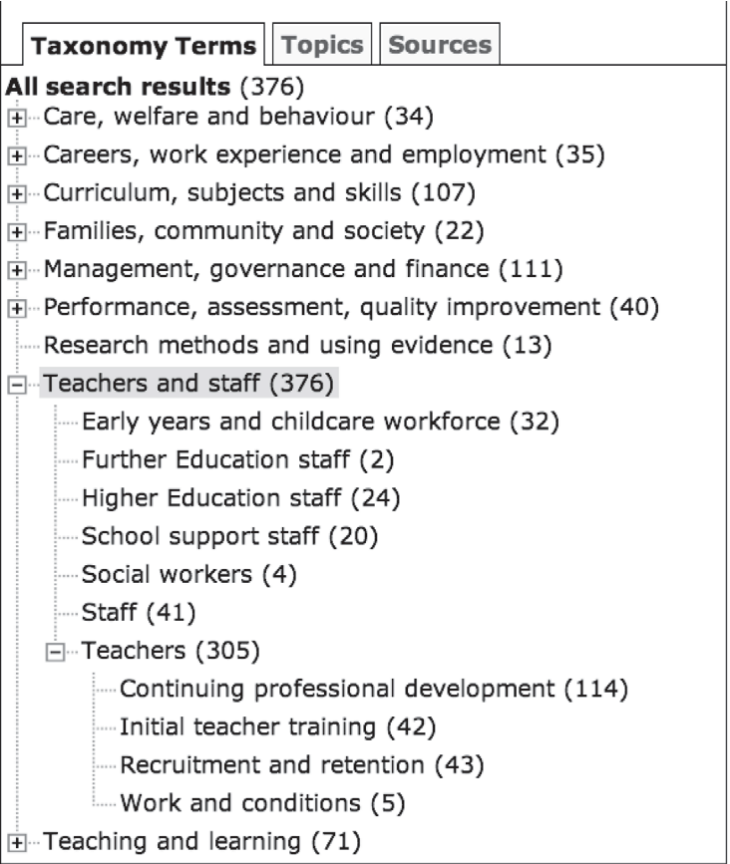
Browsing of documents using the eep taxonomy.

Figure 2 shows the display of the taxonomy in the interface after choosing the *Teachers and staff* term. There are a total of 376 documents assigned to the general *Teachers and staff* term, but there are more specific sub-terms that classify the documents at a greater level of granularity, allowing the user to view directly those documents that are most relevant to them.

In figure 2, the numbers shown on branches other than *Teachers and staff* correspond to documents that are assigned multiple labels, i.e. a label from the *Teachers and staff* branch as well as a label from one or more other branches of the taxonomy. This facilitates rapid location of very specific sets of documents. For example, documents that concern behavioural problems in the classroom can easily be located by selecting those documents that are classified under both *Care, welfare and behaviour* and *Teachers and staff*.

### (b) Machine-learning methods for document classification

Owing to the large number of documents in the eep database, and the fact that more documents are being added all the time, it is not a practical solution for all documents to be classified manually. Rather, we have used machine-learning methods to perform automatic classification of the documents in the database.

As a starting point, domain experts assigned appropriate (and mostly multiple) categories to 2157 documents, thus providing a gold-standard corpus by which to evaluate the performance of machine-learned classifiers. The features used for the automatic classification include unigrams (i.e. all unique single words that occur in a document), bigrams and trigrams (all unique groups of two or three consecutive words), as these have been demonstrated to be efficient for text categorization (Sasaki *et al*. 2009). These features were extracted from the documents following the application of the Porter stemmer (Porter 1997), which normalizes different forms of words by removing the commoner morphological and inflectional endings.

Documents in the eep database are mainly full papers or lengthy reports with an average size of 250 kB of plain text. This is in contrast to the more conventional targets of document classification, such as abstracts or newspaper articles (Sasaki *et al.* 2009). As longer documents generate a larger number of features, their automatic classification introduces a number of problems, i.e. the training of the classifiers takes much longer, the size of the classification models is much larger, and the speed of classification of each document is slower. The total number of features extracted from the documents in our experiments was 176 624 316.

Our experiments have been inspired by other studies that try to reduce the number of features required to perform classification, while also improving the classification results. Examples include Gong & Liu (2001), Bekkerman *et al*. (2003), Dhillon *et al*. (2003) and Jiang & Lee (2007). Our novel method, which is termed supervised orthogonal locality-preserving projection (SOLPP), uses information from the manually classified documents to help compute a smaller number of relevant features from the large number of original features. The SOLPP method reduced the original number of features to less than 200. Moreover, the performance of the classifier trained on this reduced set of features was superior to the performance of the classifier trained on the original features.

These results provide promising prospects for the fully automatic classification of eep documents. The small number of features needed to perform classification of documents to a reasonably high standard means that the trained classification model is reasonably small, and hence able to classify documents quickly. As future work, we plan to experiment with improving performance by including richer linguistic features such as syntactic information (Miyao & Tsujii 2008) within the classifier model.

## 6. Automatic clustering of documents

### (a) Clustering strategy

In order to overcome the problem of reviewing the potentially huge number of documents that are returned by a traditional free-text search query, the ASSIST prototype interface clusters the documents retrieved by searches according to similarities within them, and associates a readable label with each cluster. These descriptive labels can help the user to identify significant subsets within their results. Through strategic use of the navigation components, drilling down to important documents becomes increasingly easy.

The task of clustering documents presents two issues. Firstly, as the number of free-text queries that can be submitted to the system is infinite, the number of relevant clusters that should be created in response to a particular query cannot be determined in advance, and must be calculated in real time. Secondly, when the number of relevant clusters has been determined, they are only useful to users if a readable and unambiguous label can be assigned to each cluster. In the ASSIST framework, we use a search result clustering algorithm, *Lingo3G* (Osinski *et al.* 2004), which aims to address these issues.

An innovative feature of the Lingo3G algorithm is the computation of meaningful labels prior to the population of the clusters, which is carried out as follows: The most important words in documents (calculated as a function of word frequency of occurrence and document length) are firstly grouped together into *abstract concepts.* The grouping is carried out according to the cooccurrence of words in the same documents. The number of clusters created corresponds to a certain (configurable) proportion of the abstract concepts that contain the greatest number of words. Candidate labels to assign to clusters are generated by finding the longest and most frequently occurring phrases in the document snippets. Cluster labels are chosen among these candidates according to a measure of similarity against the abstract concepts. Documents are then added to clusters according to a similarity measure with the cluster label. As there are limits to the number of documents that can be processed by the algorithm within a reasonable time, processing of the search results is currently limited to the top 1000 documents returned by a search.

**Figure 3.**
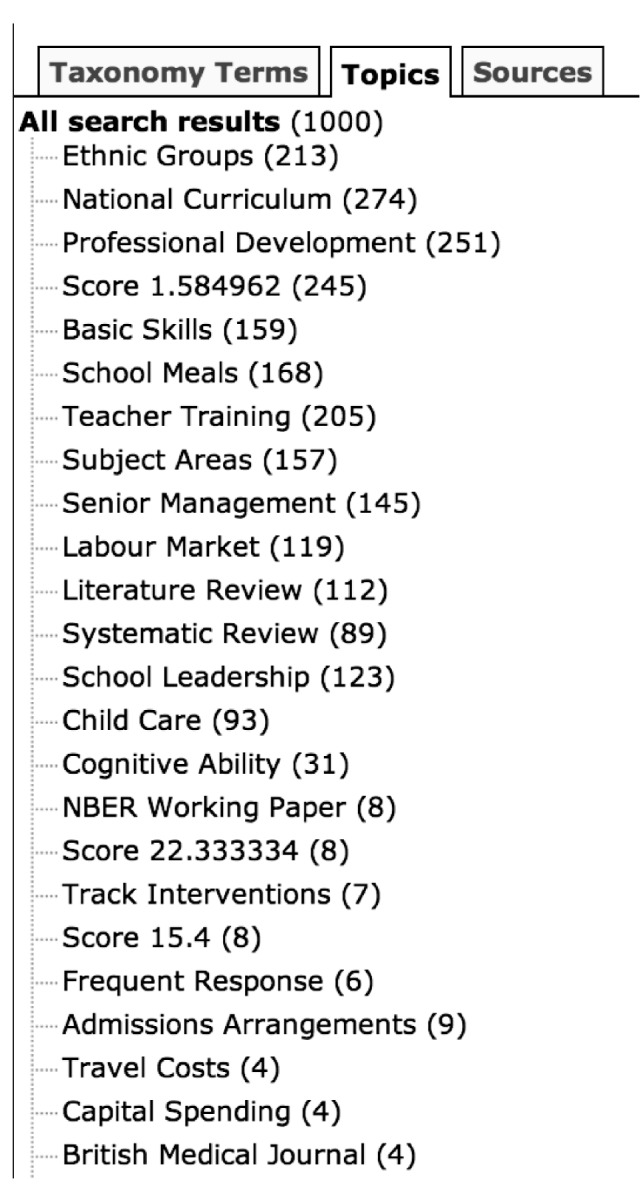
Clustering of results using the search term *English.*

Figure 3 shows the clustering results after the user has entered the search term *English.* Although this search returns a broad set of results, the automatic clustering makes the large number of returned results seem less daunting, with important subtopics clearly identified, such as English in relation to different ethnic groups, or documents relating to the teaching of English in the national curriculum or training of teachers.

## 7. User-centred evaluation—suitability and accuracy

The ASSIST project takes a user-centred design–build–evaluate approach. Although there is a large body of research on the design and evaluation of user interfaces, e.g. Shneiderman & Plaisant (2004) and Hearst (2009), most previous evaluations of text-mining systems have concentrated on the internal functionality of the system as a measure of performance.

In contrast, our evaluation is concerned more with determining the performance of the system from a user perspective and assessing how well the system actually fulfils the user’s requirements. In other words, we wish to conduct a *usability evaluation* that, according to White (2000), has the purpose of measuring the usefulness a system to its users. In the terms of the standard created by the International Organization for Standardization (ISO) and the International Electrotechnical Commission (IEC), i.e. ISO/IEC 9126-1 (2001), the question that needs to be answered is how we can reliably evaluate the *suitability* of a system. This should not be confused with evaluating the *accuracy* of a system, which only tells us how well the system can perform the tasks that it was designed to perform, without considering methods of user interaction. Good accuracy *can* contribute towards good suitability, although this does not necessarily hold—a system that is *capable* of performing *accurately* is not necessarily *suitable* if the user is unable to perform all of their required tasks in an easy and efficient manner. Thus, both accuracy and suitability can be seen as equally important.

### (a) Evaluation metrics for a text-mining-based search portal

Our evaluation framework is based largely on the dimensions outlined in King (2007). Each dimension includes positive and negative attributes established following user-oriented requirements analysis.

1. *Functionality*. This dimension concerns whether the portal meets user needs (i.e. what the portal does and not how it does it). Within this dimension, the attributes of *suitability* (provision of an appropriate set of functions for specified tasks and user objectives), *accuracy* (whether the software conforms to the requirements), *interoperability* (embedding within other systems) and *compliance to standards* have also been assessed by end users, partners and the host organization of the eep.
2. *Reliability*. This deals with issues such as how well the portal can deal with heavy and unpredictable user demand, or poorly formed/malicious queries.
3. *Usability*. This dimension is concerned with whether or not users are able to use the functions of the portal to achieve their objectives. Usability is measured using four different attributes. The first three of these, *understandability*, *learnability* and *operability*, are addressed by giving users specific tasks to undertake using the portal (e.g. locating information about particular topics), and then asking them about how they approached the task. The other usability attribute is the *attractiveness* of the interface.
4. *Efficiency.* This deals with both how quickly users are able to accomplish their objectives, as well as the demands the system places on the computational infrastructure of the host organization.
5. *Maintainability*. This concerns the ease with which the software can be corrected, improved or adapted. This is mainly carried out internally and relates to error analysis, modification of portal functionalities based on integration of new text-mining tools, stability of the portal, etc.

Using this methodology, the evaluation combines a selection of quantitative and qualitative approaches, providing a practical solution to the tasks for which no realistic gold-standard assessment can be made.

### (b) Evaluation results

The results of the evaluation are currently being analysed, but we are able to present some emerging findings in this section, in terms of what has been learned about the strengths, weaknesses and potential improvements of the three key features of the portal, i.e. automatic document clustering, related topics and related documents (generated according to the results of analysis by TerMine), and automatic classification of documents according to the taxonomy. These interim findings are based on 25 interviews (either face-to-face or via telephone) and 68 online questionnaires completed by current users of the portal. Participants were comprised of approximately equal numbers of education practitioners, researchers and information scientists.

#### (i) Automatic document clustering

*Strengths*. Approximately 74 per cent of respondents either agreed or strongly agreed that automatic document clustering was a useful feature to refine search results (‘I see that it enables you to sub-select something like ‘basic skills’ which would be useful’). The usefulness of automatic clustering, however, was shown to depend on: (i) a desire to browse (‘I can’t see I’d use this feature much because I tend to come to databases with a specific task, not to browse generally’) and (ii) a recognition of one’s interests in the topics available.

*Weaknesses.* Two main problematic areas were reported: accuracy and understandability. With respect to accuracy, evaluation participants reported that 51 per cent of the automatically generated clusters were useful, but 12 per cent (standard deviation s.d. 10%) thought that they were not relevant. More of an issue for users, however, was the fact that the automatic clustering function was not easy to understand. Even after using it, interviewees were not able to explain the exact function and nature of the clusters. Typical responses were ‘I’m not sure where they come from’, ‘are they linked to the taxonomy terms?’ and ‘another way of sorting information’.

*Improvements.* Suggested improvements included the provision of clear guidance as to the differences between using clusters and taxonomy terms to refine search results, and also the ability for users to select several topics simultaneously to further narrow down results.

#### (ii) Related documents and topics

*Strengths.* While the usefulness and accuracy of these features was questioned by users, they seemed relatively easy for people to use and understand, at least at a basic level. Having experimented with ‘related documents’, for example, most interviewees had a sense of what it was providing, if not a clear understanding of how it worked. One interviewee compared this feature to ‘a report bibliography, i.e. giving further/other references’.

*Weaknesses.* Users were slightly less convinced about the utility of the related documents/topics feature than other portal features. Several people felt that they may cause ‘information overload’ and ‘may end up pulling users away from their search’. However, some users made the point that they could be useful ‘if you don’t know what you are looking for’ or ‘want to browse for related peripheral documents’. There were also some concerns about accuracy—on average, 53 per cent (s.d. 33%) of the ‘related documents’ were considered appropriate and about half of the ‘related topics’ (49% s.d. 24%) were thought to be useful. Furthermore, it was clear that some users did not find it easy to understand the purpose of ‘related topics’. Some said they simply ‘can’t understand these’ or made fairly vague references to ‘pulling out related items’.

*Improvements.* In light of the issues raised above, there were calls for clearer signposting for both ‘related documents’ and ‘related topics’, as well as a better explanation about the purpose and basis of these functions.

#### (iii) Automatic document classification according to taxonomy

*Strengths.* Automatic document classification received the strongest support in terms of being a useful feature of the portal (approx. 85% agreeing or strongly agreeing), as backed up by the following comment: ‘The taxonomy terms are very useful in giving me a way to break down the search results by other areas. I like that as the taxonomy terms are gateways into the information’. In terms of understandability, most interviewees were able to determine what the taxonomy terms are, what they do and how they can be used. Descriptions included ‘nine key terms under which documents will be clustered’ and ‘pre-defined terms that allow things to be put in categories’. However, it should be noted that familiarity with ‘the sub-divisions of Children’s Services’ and ‘library-type classification systems’ seemed to help with understanding the taxonomy. On average, 59 per cent (s.d. 25%) of the automatically assigned taxonomy terms were deemed to be correct.

*Weaknesses.* Selecting a taxonomy term also causes the documents within that branch of the taxonomy to be clustered and assigned topics by the Lingo3G clustering engine. As there are limits to the number of documents that can be processed by the clustering engine within a reasonable amount of time, each search returns a limit of 1000 documents. This limit works admirably for free-text searches, where the most relevant material is assumed to be at the top of the list. However, the results of browsing the taxonomy tree cannot be ordered in terms of their relevance. There is therefore a danger that the search is truncated arbitrarily and a possibility that results that are relevant to the user are lost. Users also questioned the accessibility of the taxonomy to non-specialist users.

*Improvements.* Suggested improvements included displaying the taxonomy terms in an un-expanded format, ensuring a better explanation of the relationship (if any) between the taxonomy terms and the free-text search and providing more guidance on the basis and function of the taxonomy.

## 8. Conclusions and further work

Text-mining services have been used to enhance search and discovery options for the UK eep. Combinations of metadata enhancement, improved browsing and navigation, alongside alternative views of resources, have all strengthened the overall proposition of the portal. Particular features include the automatic classification of lengthy documents and reports (as opposed to only abstracts) according to a custom-built, domain-specific taxonomy, automatic grouping of documents into clusters that are generated on demand according to the contents of the retrieved documents, and automatic identification of key terms within documents, which facilitates quick scanning of documents, as well as allowing closely related documents to be identified. Collectively, these features provide the ability to search for relevant information in a more timely and efficient manner than was previously possible. The enhanced features of the portal provide the potential to revolutionize education practice that, owing to time limitations, sometimes does not take account of research evidence at all.

Regular and continued user engagement during the lifetime of the project has led to a significant service exemplar of the applications and benefits of text mining within the social sciences. Rigorous quality assurance and comprehensive evaluation strategies have been used to ensure the tools meet the needs of the eep stakeholders, and we anticipate that this will be extended further to support a framework for the wider evaluation of text-mining components. The interim evaluation results suggest that users largely have positive attitudes towards the enhanced searching facilities offered by the new interface, although certain problems, such as the accuracy of the search results, still need to be addressed. It also seems clear that the interface needs to provide more detailed guidance on the usage and purpose of each of the new technologies.

As we further expand upon the work of the ASSIST project, opportunities to reflect upon the outputs of the eep and related projects have highlighted several strands of potential future development. Automatic identification of *named entities* (NEs), such as names of people, places or organizations, dates, job titles, etc., could not only provide enhanced visualization of documents, but could also facilitate more sophisticated search functionality. Through the provision of operators that allow the specification of particular entity types as part of a query, it is possible to distinguish, for example, those documents discussing the city of *London* from resources written by or discussing the person *John London*. Queries could also be performed that, for example, list all of the organizations discussed within resources describing a particular area of research.

The benefits of the recognition of different types of NE within a different domain has been illustrated by Kleio (Nobata *et al*. 2008) (http://www.nactem.ac.uk/software/kleio/), another of NaCTeM’s tools, designed to process biomedical documents. Names of proteins, such as *cat*, can be ambiguous with common English words. A traditional search engine will return all the documents that mention either the protein or the animal, resulting in more than 60 000 being returned from a search across the whole of MEDLINE (an online database of citations and abstracts from medical journals available at http://www.ncbi.nlm.nih.gov/pubmed/). In Kleio, it is possible to search only for documents containing instances of the word *cat* that have been identified as NEs of type protein, using a query modifier to indicate the type of entity to search for, as in ‘*Protein : cat’*, which returns only 237 documents.

Other disciplinary domains could clearly benefit from services similar to those that have been created as extensions to the eep, either involving further subject customization or through the integration of complementary components. Given the wider themes surrounding repositories and metadata in the community, the role of text mining is destined to become increasingly important.
